# Low-quality employment trajectories and mental health disorders among Swedish and migrant workers

**DOI:** 10.1093/eurpub/ckac129.615

**Published:** 2022-10-25

**Authors:** R Pollack, B Kreshpaj, J Jonsson, T Bodin, V Gunn, C Orellana, P Östergren, C Muntaner, N Matilla-Santander, PWR Consortium

**Affiliations:** 1 MRC/CSO Social and Public Health, University of Glasgow, Glasgow, UK; 2 Unit of Occupational Medicine, Karolinska Institutet, Stockholm, Sweden; 3 Centre for Occupational and Environmental Medicine, Stockholm Region, Stockholm, Sweden; 4 MAP Centre for Urban Health Solutions, Unity Health Toronto, Toronto, Canada; 5 Lawrence S. Bloomberg Faculty of Nursing, University of Toronto, Toronto, Canada; 6 Social Medicine and Global Health, Lund University, Lund, Sweden; 7 Dalla Lana School of Public Health, University of Toronto, Toronto, Canada

## Abstract

**Aim:**

This study aims to examine the effects of low-quality employment trajectories on severe common mental disorders (CMD) according to Swedish and foreign background.

**Methods:**

This is a longitudinal study based on Swedish population registries (N = 2,703,687). Low- and high-quality employment trajectories observed across five years (2005-2009) are the exposure with severe CMD as outcome (2010-2017). Adjusted hazard ratios (HR) were calculated using Cox regression stratified according to background (first-generation (i) EU migrants, (ii) non-EU migrants, (iii) second-generation migrants, (iv) Swedish-born with Swedish background) and sex. The reference group were Swedish-born with Swedish background in a Constant high-quality employment trajectory.

**Results:**

Second-generation migrants had an increased risk of CMD compared to Swedish-born with Swedish background when following low-quality employment trajectories (e.g., male in Constant low-quality HR: 1.53, 95% CI: 1.41-1.68). Female migrant workers, especially first-generation from non-Western countries in low-quality employment trajectories (e.g., Constant low-quality HR: 1.65, 95% CI:1.46 - 1.87), had a higher risk of CMD compared to female Swedish-born with Swedish background. The confidence interval for CMD risk showed little differences between migrant groups (1st and 2nd generation) compared to the reference group.

**Conclusions:**

Low-quality employment trajectories appear to be determinants of risk for CMD, having a differential impact according to background of origin and sex. We observe a higher risk for severe CMD across migrant groups, especially second-generation migrants, compared to Swedish-born with Swedish background. Further qualitative research is recommended to understand the mechanism behind the differential mental health impact of low-quality employment trajectories according to foreign background.

**Key messages:**

• First and second-generation migrants in low quality employment have higher risk of severe common mental disorders compared to Swedish born with Swedish background workers in low quality employment.

• Policies targeting working conditions in low-quality employment and promoting workers mental well-being are essential to reduce this higher risk for developing CMD, especially for migrant populations.

